# Inhibition of Tcf-4 Induces Apoptosis and Enhances Chemosensitivity of Colon Cancer Cells

**DOI:** 10.1371/journal.pone.0045617

**Published:** 2012-09-24

**Authors:** Jiang Xie, De-Bing Xiang, Hong Wang, Cong Zhao, Jie Chen, Feng Xiong, Ting-Yu Li, Xiao-Lei Wang

**Affiliations:** 1 Children’s Hospital, Chongqing Medical University, Chongqing, China; 2 The Third People’s Hospital of Chengdu, Sichuan, China; 3 Cancer Center, Jiangjin Central Hospital, Chongqing, China; 4 Department of Laboratory Medicine, Key Laboratory of Diagnostic Medicine (Ministry of Education), Chongqing Medical University, Chongqing, China; Vanderbilt University Medical Center, United States of America

## Abstract

Aberrant activation of β-catenin/Tcf-4 signaling has been implicated in human carcinogenesis, including colorectal cancer. In this study, we compared the effects of Tcf-4 knockdown with β-catenin knockdown on cell proliferation, apoptosis, and chemosensitivity in SW480 and HCT116 colon cancer cells using adenoviral vector-mediated short hairpin RNA (shRNA). Our results show that, compared to β-catenin knockdown, Tcf-4 knockdown more effectively inhibited colony formation, induced apoptosis, and increased 5-FU and oxaliplatin-mediated cytotoxicity in colon cancer cells. We further investigated the mechanisms involved in the different efficacies observed with β-catenin and Tcf-4 knockdown in colon cancer cells. FOXO4 is a member of the subfamily of mammalian FOXO forkhead transcription factors and plays a major role in controlling cellular proliferation, apoptosis, and DNA repair. Our data showed that the protein level of FOXO4 did not change after treatment with both β-catenin and Tcf-4 shRNA. However, β-catenin shRNA was found to increase the accumulation of phosphorylated FOXO4 S193 and decrease the expression of FOXO target genes p27Kip1 and MnSOD, whereas Tcf-4 shRNA showed the opposite effect. Therefore, compared to β-catenin knockdown, Tcf-4 knockdown shows better efficacy for inhibiting proliferation and inducing apoptosis of colorectal cancer cells, which may be related to increased FOXO4 transcriptional activity. These results suggest that Tcf-4 is an attractive potential therapeutic target for colorectal cancer therapy.

## Introduction

The canonical Wnt signaling pathway plays a central role in numerous cellular processes, from embryonic development to adult tissue homeostasis [1]. The activity of this signaling pathway is determined by the amount of β-catenin present in the cytoplasm. In the absence of Wnt signaling, cytoplasmic β-catenin is normally maintained at low levels through continuous ubiquitin-proteasome-mediated degradation of β-catenin, which is regulated by a destruction complex composed of adenomatous polyposis coli (APC), glycogen synthase kinase-3β (GSK-3β), Axin/Conductin, Casein Kinase 1α (CK1α), and other proteins that mediate these biochemical reactions. Binding of Wnt proteins to the cell surface receptor complex Fz/LRP facilitates the phosphorylation of the cytoplasmic tail of LDL receptor-related protein (LRP) cytoplasmic tail by GSK-3β [2]. This triggers the interaction of the Fz/LRP complex with Dishevelled (Dsh) and Axin, which leads to the inactivation of the destruction complex, resulting in the accumulation of non-phosphorylated β-catenin in the cytoplasm. The accumulated β-catenin then translocates into the nucleus and binds to Tcf/Lef transcription factors to regulate downstream target genes, such as c-Myc and Cyclin D1 [3]–[5].

Aberrant Wnt/β-catenin signaling has been reported to contribute to various human diseases, including colorectal cancer (CRC) [6], [7]. The APC gene or the GSK-3β phosphorylation site within exon 3 of the *β-catenin* gene (*CTNNB1*) is mutated in many cancer cells, including CRC, resulting in the activated transcriptional activity of β-catenin/Tcf signaling [8]. Moreover, nuclear translocation of β-catenin in CRC is significantly associated with tumor progression and poor survival [9], [10]. Therefore, control of β-catenin and/or control of its downstream target gene expression represents an ideal target for cancer therapeutics and chemoprevention [11], [12], [13].Van de Wetering *et al*. reported that knockdown of β-catenin by small interfering RNAs (siRNAs) or knockdown of TCF-4 by dominant negative TCF-4 (dnTCF) efficiently inhibited the activity of the TCF-reporter TOPFlash and induced cell cycle arrest and growth arrest in Ls174T colon cancer cells [14], [15]. However, Tang *et al.* reported that knockdown of TCF-4 by siRNAs increased cell growth in DLD-1 colon cancer cells [16]. These discrepancies suggest that different cell lines might respond differently to TCF-4 knockdown.

FOXO4 is one member of the subfamily of mammalian FOXO forkhead transcription factors [17] that are important in a variety of processes, including cellular proliferation, differentiation, apoptosis, DNA repair, and protection from stress [18]. It is an AKT downstream target and becomes phosphorylated on three highly conserved serine and threonine residues (Thr-28, Ser-193, and Ser-258) upon PKB/AKT activation. Recent studies have shown that β-catenin binds to the FOXO transcription factor, which plays a tumor suppressor role in a variety of cancers. β-catenin binds to FOXO and enhances the transcriptional activity [19]. Moreover, cGMP-dependent protein kinase (PKG) inhibits TCF signaling in colon cancer cells by blocking β-catenin expression and activating FOXO4 [20]. Therefore, β-catenin appears to serve a dual effect by balancing positive (through Tcf-4) and negative (through FOXO4) regulation of cell proliferation and apoptosis.

In this study, we hypothesized that the downstream transcription factor Tcf-4 is a more promising therapeutic target than β-catenin for treating CRC. Our aim was to compare the effects of Tcf-4 knockdown and β-catenin knockdown on cell proliferation, apoptosis, and chemosensitivity in the SW480 (mutant *APC*, wild-type *CTNNB1*) and HCT116 (mutant *CTNNB1*, wild-type *APC*) colon cancer cell lines using short hairpin RNA (shRNA). We show that compared to β-catenin knockdown, Tcf-4 knockdown significantly inhibits cell proliferation, induces cell apoptosis, and enhances the chemosensitivity of colon cancer cells through the upregulation of FOXO4 transcriptional activity.

## Materials and Methods

### Cell Culture and Reagents

SW480 and HCT116 cells were purchased from the American Type Culture Collection (Manassas, VA, USA) and maintained in RPMI1640 supplemented with 10% fetal bovine serum (FBS) and 100 U/ml of penicillin and streptomycin under standard culture conditions. Oxaliplatin, 5-fluorouracil (5-FU), and methyl thiazolyl tetrazolium (MTT) were purchased from Sigma-Aldrich (St Louis, MO, USA). The plasmid pDC316-EGFP-U6 was provided by Vector Gene Technology Company Limited (VGTC, Beijing, China) and the plasmid pBHGloxΔE1, 3Cre was provided by Microbix Biosystems, Inc. (Microbix, Toronto, Canada).

### Recombinant Adenovirus Vectors

#### On the basis of cDNA sequences (*β-catenin* GenBank accession no

NM_001098209, *Tcf-4* GenBank accession no. NM_030756), one specific pair of oligonucleotides with a short hairpin and its negative control sequence were designed and synthesized, and then inserted into a small shuttle plasmid pDC316-EGFP-U6 at the BamH I and Hind III restriction enzyme sites. Oligonucleotides were designed with the following primers [21]:

#### Forward β-catenin shRNA primer

′-GATCCCGTGGGTGGTATAGAGGCTCTTCAAGAGAGAGCCTCTATACCACCCACTTTTTGGAAA-3′, reverse β-catenin shRNA primer: 5'-AGCTTTTCCAAAAAGTGGGTGGTATAGAGGCTCTCTCTTGAAGAGCCTCTATACCACCCACGG-3′;

### Forward Tcf-4 shRNA Primer

5'-GATCCCCGGAGCGACAGCTTCATATGTTCAAGAGACATATGAAGCTGTCGCTCCTTTTTGGAAA-3′, reverse Tcf-4 shRNA primer:

5'-AGCTTTTCCAAAAAGGAGCGACAGCTTCATATGTCTCTTGAACATATGAAGCTGTCGCTCCGGG-3;

#### Control shRNA: forward direction

5′-GATCCCCCAGTAACTGAATAGCTACCTTCAAGAGAGGTAGCTATTCAGTTACTGTTTTTGGAAA-3′, reverse direction. 5′-AGCTTTTCCAAAAACAGTAACTGAATAGCTACCTCTCTTGAAGGTAGCTATTCAGTTACTGGGG-3′. The control shRNA did not have homology to any relevant human genes. All constructs were verified by DNA sequencing. The resulting shuttle plasmids were co-transfected with the adenovirus rescue plasmid pBHGloxΔE1, 3Cre into 293 cells to acquire recombinant adenovirus. Recombinant adenovirus efficiency was detected by examining the cytopathic effect and EGFP expression in the cells. The adenovirus titers were measured after amplification and purification using TCID50 assays.

### Colony Formation Assay

SW480 cells were infected with recombinant adenoviruses for 90 min, and after 24 h, they were seeded at 300 cells/well in 6-well plates and allowed to attach for 24 h. After incubation, the medium was changed and the plates were incubated for an additional 10 days under the same culture conditions. Colonies were fixed and stained with 0.1% crystal violet in 100% ethanol and then counted. Clones of at least 50 cells were counted as one colony.

### Cytotoxicity Assays

SW480 cells were infected with the recombinant adenoviruses for 90 min, and after 24 h, they were plated in 96-well plates at 4000 cells/well. Following 24 h culture, cells were treated with the indicated concentrations of 5-FU or oxaliplatin for 72 h. Then, 20 µL MTT (5 g/L) was added to each well and incubated for an additional 4 h. The culture media were then discarded, 0.15 mL dimethyl sulfoxide (DMSO) was added, and the plates were incubated for an additional 10 min with vibration. The absorbance was measured at 490 nm using a microplate reader (model 550, Bio-Rad, USA).

### Apoptosis Assay

SW480 cells were plated in six well plates at a density of 0.5×10^6^ cells/well. Twenty-four hours after plating, cells at 70% confluency were infected with adenoviruses for 90 min and then washed to remove the adenoviruses. Following an additional 24 h culture, the apoptotic index was assessed by flow cytometry using Annexin-V-FITC kit as previously described [22].

### Western Blot Analysis

Cells were harvested and total proteins were extracted with RIPA buffer containing protease inhibitors. Western blot was carried out as previously described [22]. Briefly, equal protein aliquots (50 µg) in each sample were resolved by 10 or 12% sodium dodecyl sulfate polyacrylamide gel electrophoresis (SDS-PAGE) and the proteins were transferred onto polyvinylidene difluoride (PVDF) membranes. After blocking with 5% non-fat dried milk, the membranes were incubated with antibodies to β-catenin (1∶5,000), Tcf-4 (1∶1,000), c-myc (1∶2000), cyclin D1 (1∶2000), FOXO4 (1∶1000), FOXO4 S193 (1∶500), p27Kip1 (1∶2000), MnSOD (1∶2000), Caspase-3 (1∶500), and β-actin (1∶3000). The membranes were then incubated with a horseradish peroxidase-conjugated secondary antibody (1∶2000) (Pierce, Rockford, IL, USA). The proteins were detected with an enhanced chemiluminescence detection system (Pierce), and light emission was captured on Kodak X-ray film.

### Transfection and Reporter Gene Assay

For measurements of β-catenin/Tcf-4 transcriptional activity, a pair of luciferase reporter plasmids (TOPflash/FOPflash; Upstate Biotechnology) were used. The pRL-TK luciferase reporter gene plasmid (Promega) was co-transfected to normalize for transfection efficiency. Transient transfection was performed using Fugene 6 (Roche, Indianapolis, IN, USA) according to the manufacturer’s instructions. The cells were infected with the recombinant adenoviruses for 90 min, and after 24 h, were subsequently co-transfected with TOPflash luciferase reporter (or mutant control FOP flash vector) and pRL-TK. After an additional 24 h incubation, cells were harvested for luciferase activity measurement using the dual-luciferase reporter assay system (Promega). Three replicate experiments were performed.

### Statistical Analysis

Data were expressed as the mean ± standard deviation (s.d.). The statistical significance of differences was determined by one way analysis of the variance (ANOVA) using SPSS v12.0 software (SPSS, Chicago, Illinois, USA). A value of *P*<0.05 was considered statistically significant.

## Results

### 1. Knockdown of Tcf-4 or β-catenin by Adenovirus-mediated Transduction of shRNA

**Figure 1:**
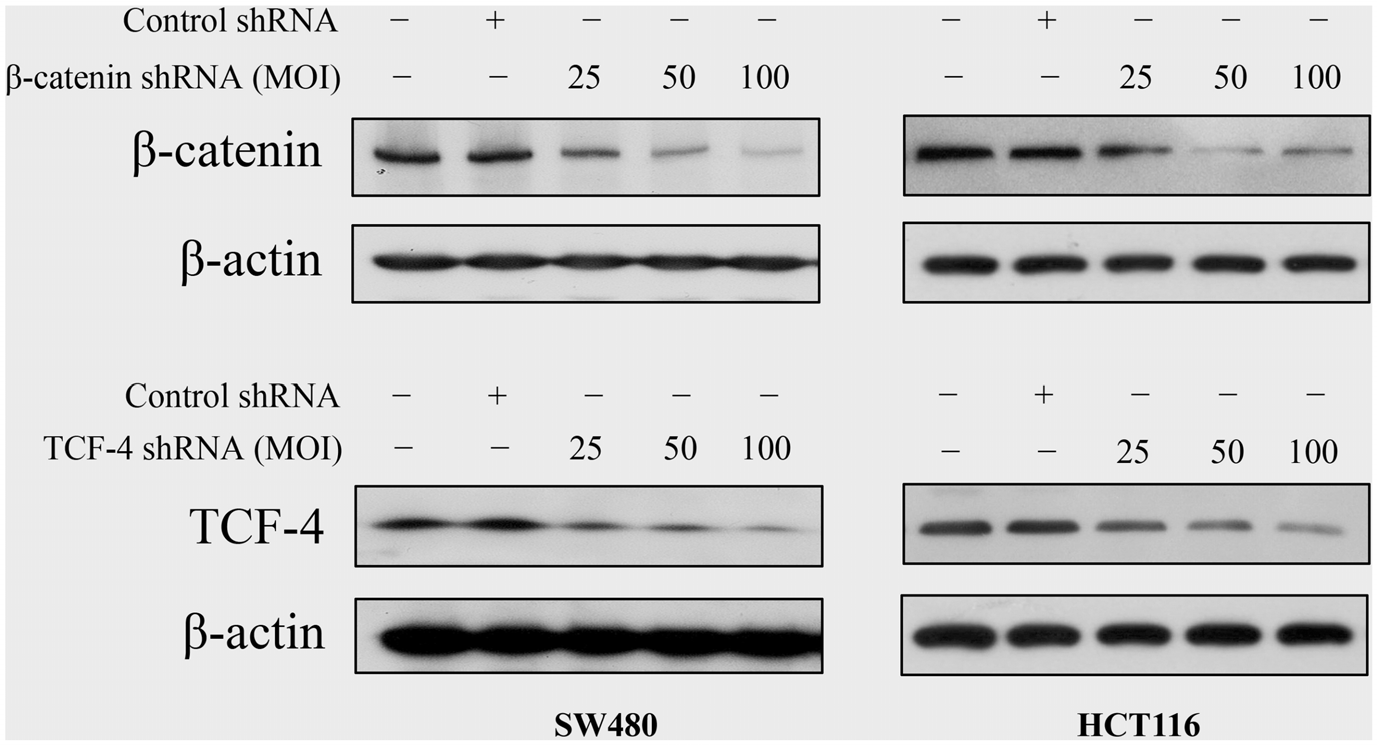
Effect of adenovirus-mediated transduction of shRNA on the expression of β-catenin and Tcf-4 in colon cancer cells. SW480 and HCT116 cells were treated with different multiplicity of infection (MOI) of adenovirus carrying shRNA. Samples were collected at 48 h post-infection. Western blot analysis of cell lysates for the protein expression of β-catenin (a) and Tcf-4 (b).

To knockdown the expression of Tcf-4 or β-catenin, adenoviral vectors were generated to express a shRNA targeting Tcf-4 or β-catenin. Western blot analysis revealed a dose-dependent decrease in Tcf-4 or β-catenin protein expression at 48 h post-infection with the respective adenoviral vectors in SW480 and HCT116 cells, while no change in the protein expression was observed after infection with an adenovirus containing scrambled shRNA (Fig. 1).

### 2. Tcf-4 Knockdown Suppresses Wnt Signaling

**Figure 2:**
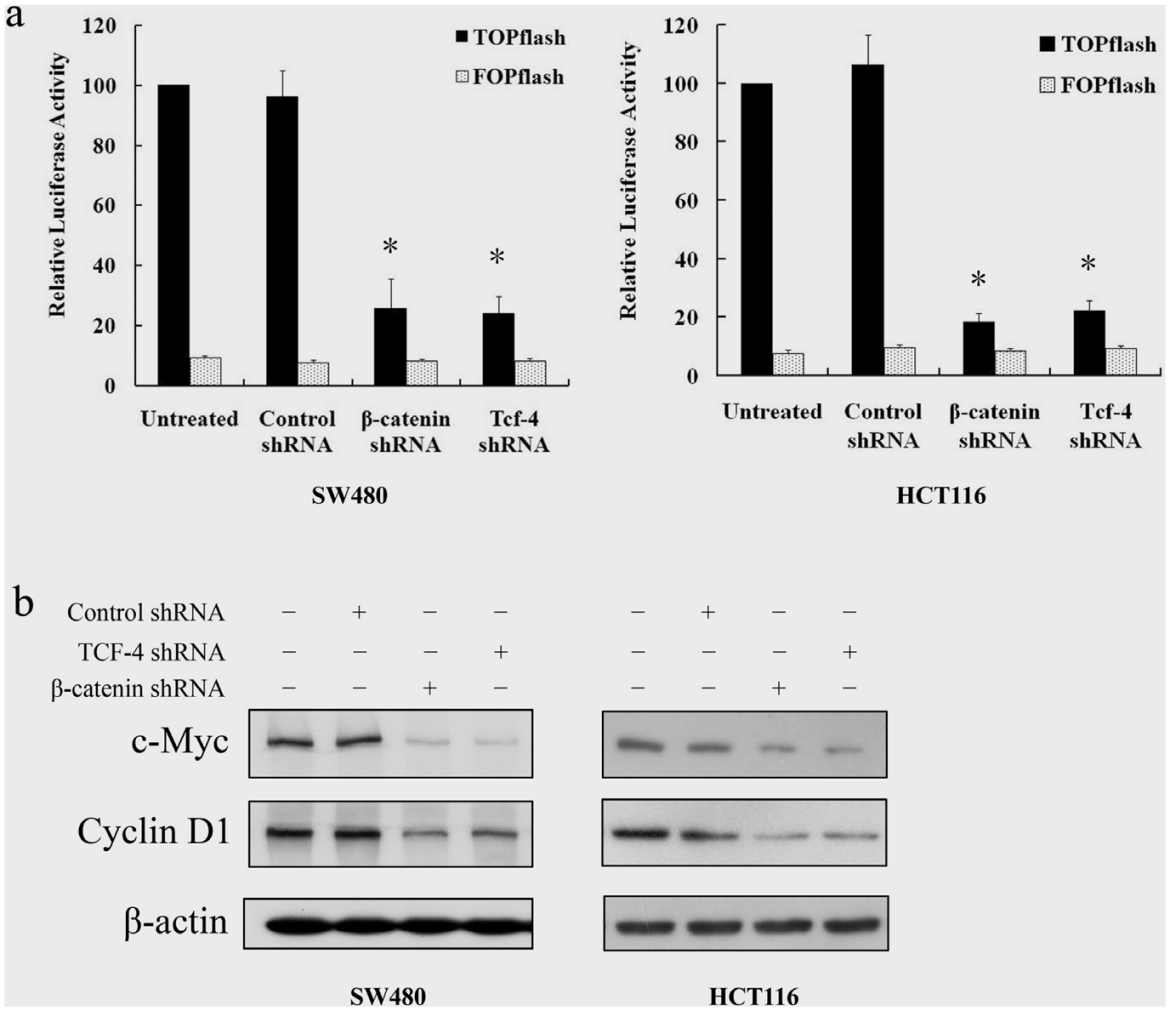
Tcf-4 knockdown suppresses Wnt signaling in colon cancer cells. SW480 and HCT116 cells were treated with adenoviruses (50 MOI) carrying shRNA. **a**, At 24 h post-infection, the cells were cotransfected with reporter genes harboring Tcf-4 binding sites (TOPflash) or a mutant Tcf-binding site (FOPflash), respectively, together with pRL-TK. Luciferase activity was determined 24 h post-transfection, normalized against values for the corresponding pRL-TK activity. Values represent means ± s.d. of three independent experiments. *P<0.01 versus control shRNA. **b**, At 48 h post-infection, cells were harvested and protein expression was determined by western blot.

SW480 and HCT116 cell lines have constitutively active β-catenin/Tcf-4 transcriptional activity. Therefore, cells were transiently transfected with a TCF reporter plasmid, TOPflash, which consists of three TCF binding sites upstream of a minimal tk promoter and the luciferase open reading frame, or control plasmid, FOPflash, which is identical to TOPflash except that it contains mutant inactive TCF binding sites. Figure 2a showed that both β-catenin shRNA and Tcf-4 shRNA markedly suppressed endogenous β-catenin/Tcf-4 transcriptional activity compared to the control shRNA in both SW480 and HCT116 cell lines.

*Cyclin D1* and *c-Myc* are known β-catenin/Tcf-4 target genes, and therefore we also investigated the protein expression of cyclin D1 and c-Myc by western blot. Figure 2b shows that β-catenin shRNA or Tcf-4 shRNA treatment resulted in a marked decrease in Cyclin D1 and c-Myc protein in both SW480 and HCT116 cells.

### 3. Tcf-4 Knockdown Enhances FOXO4 Activity

**Figure 3:**
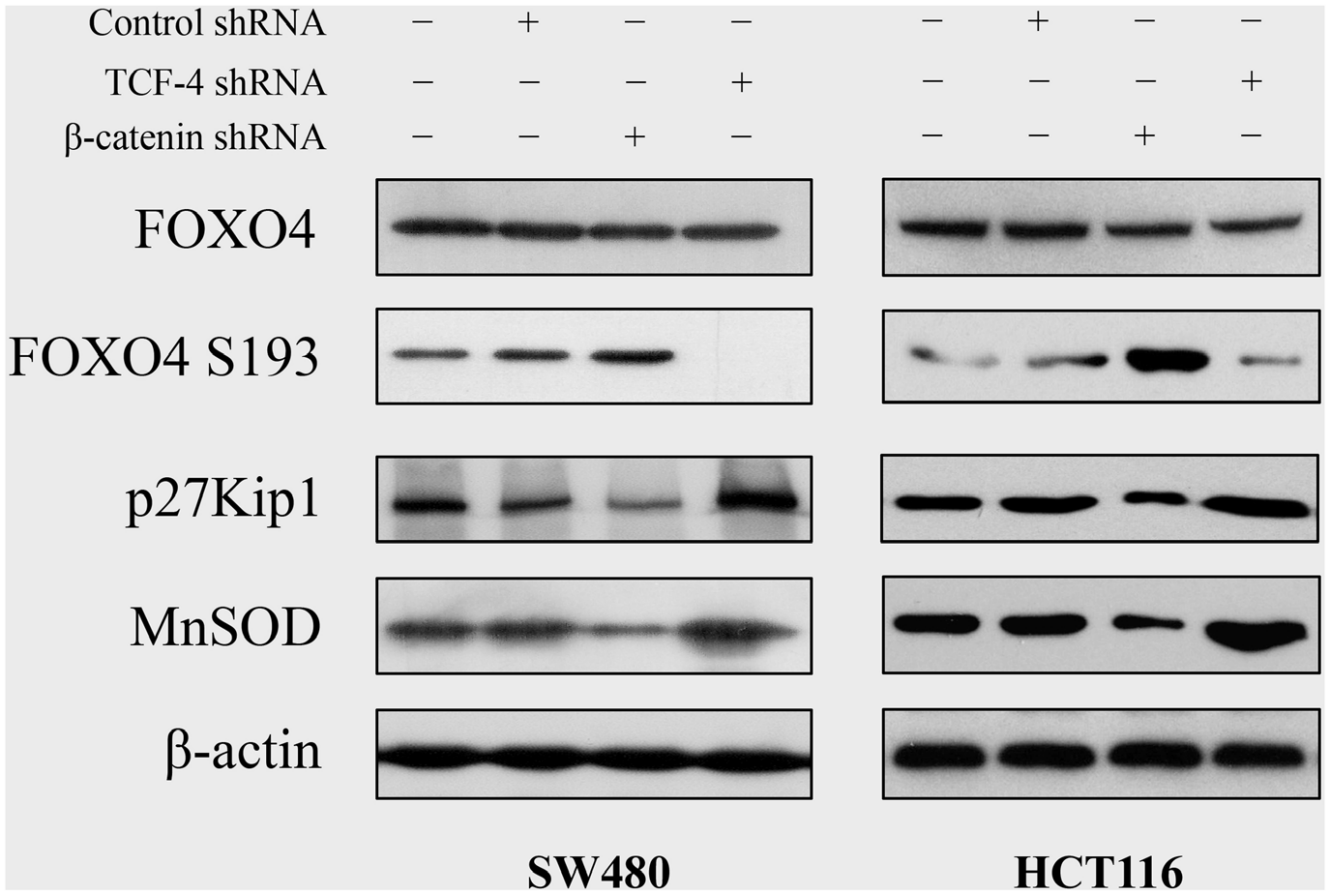
Tcf-4 knockdown enhances FOXO4 activity in colon cancer cells. SW480 and HCT116 cells were treated with adenoviruses (50 MOI) carrying shRNA. Samples were collected at 48 h post-infection. The protein level of FOXO4, phosphorylated FOXO4 S193, p27Kip1, and MnSOD was determined by western blot.

To analyze the protein level and activation of FOXO4 protein, SW480 and HCT116 cells were grown for 48 h after infection with adenoviruses, and the protein level was assessed by western blot. The protein level of FOXO4 was not significantly changed after treatment with β-catenin shRNA or Tcf-4 shRNA (Fig. 3). However, the protein level of phosphorylated FOXO4 S193 was markedly increased after treatment with β-catenin shRNA, and decreased after treatment with Tcf-4 shRNA (Fig. 3). The expression levels of FOXO target genes p27Kip1 and MnSOD were markedly decreased after treatment with β-catenin shRNA and increased after treatment with Tcf-4 shRNA (Fig. 3).

### 4. Tcf-4 Knockdown Inhibits Cell Proliferation and Induces Cell Apoptosis in Colorectal Cancer Cells

**Figure 4:**
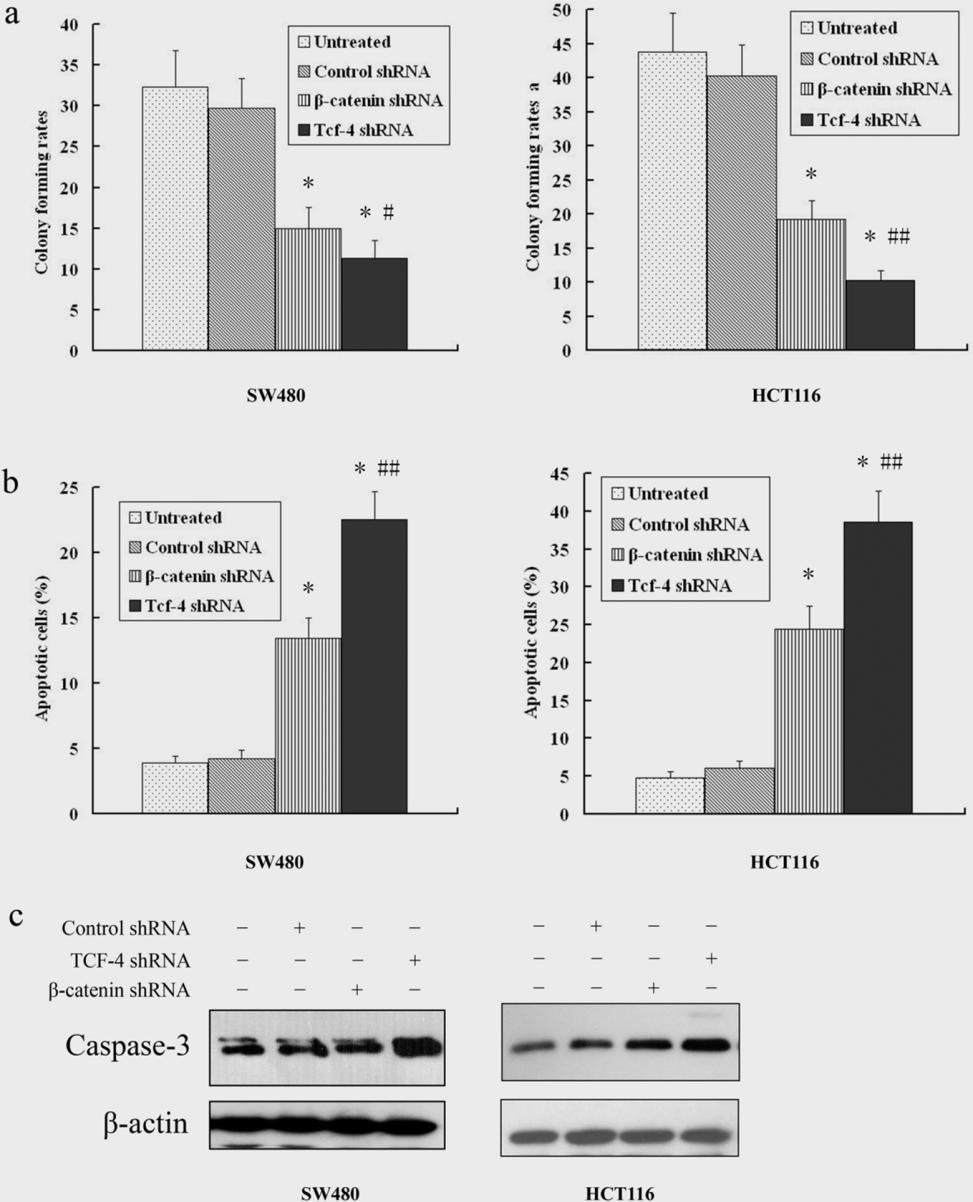
Tcf-4 knockdown is more effective at inhibiting cell proliferation and inducing apoptosis than β-catenin knockdown in colon cancer cells. Cells were treated with adenoviruses (50 MOI) carrying shRNA for 24 h. **a,** Cell proliferation was determined using a colony formation assay. **b,** Cell apoptosis was determined using Annexin-V-FITC staining. **c**, The expression of proapoptotic enzyme caspase-3 was determined by western blot. Each data point represents the mean ± s.d. of three or more independent determinations. *P<0.01 versus control shRNA; ^#^ *P*<0.05 versus β-catenin shRNA; ^##^ *P*<0.01 versus β-catenin shRNA.

We next investigated the effect of Tcf-4 knockdown on cell proliferation and apoptosis in SW480 and HCT116 cells. Cell proliferation was determined using a colony formation assay. As shown in Figure 4a, Tcf-4 knockdown induced a significantly stronger inhibition of cell proliferation compared to β-catenin knockdown in both SW480 and HCT116 cells (p<0.05, p<0.01, respectively). To investigate whether Tcf-4 shRNA-mediated growth inhibition is associated with apoptosis, treated and untreated cells were analyzed by flow cytometry. As shown in Figure 4b, both β-catenin shRNA and Tcf-4 shRNA induced a significant increase in the number of apoptotic cells compared to untreated cells and control shRNA treated groups (p<0.01), and Tcf-4 shRNA induced a greater number of apoptotic cells than β-catenin shRNA (p<0.01). The proapoptotic enzyme caspase-3 is activated at a point of convergence between the intrinsic and extrinsic apoptosis induction pathways [23]. Therefore, we examined caspase-3 cleavage as a marker of apoptosis. Consistent with the flow cytometry findings, Tcf-4 shRNA induced a higher increase in caspase-3 cleavage compared to β-catenin shRNA (Fig. 4c).

### 5. Tcf-4 Knockdown Enhances Chemosensitivity in Colorectal Cancer Cells

**Figure 5:**
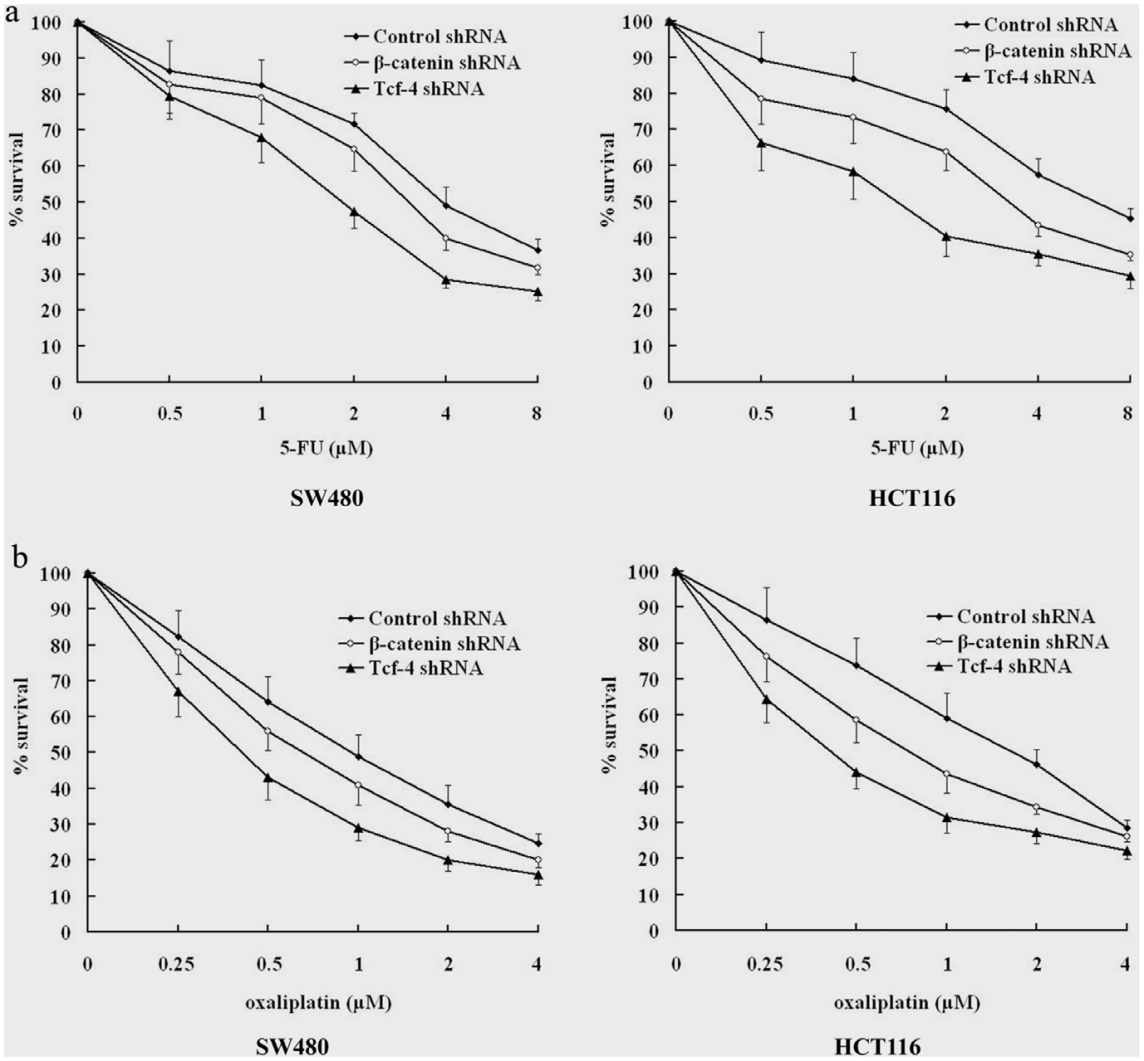
Tcf-4 knockdown is more effective at enhancing chemosensitivity than β-catenin knockdown in colon cancer cells. Cells were infected with adenoviruses (50 MOI) carrying shRNA; 48 h post-infection, cells were treated with 5-FU or oxaliplatin at various concentrations for 72 h. The cell viability was determined using an MTT assay. Bar graphs represent the mean values of triplicate determinations ± s.d.

SW480 and HCT116 cells were pretreated with the recombinant adenoviruses carrying the respective shRNA and were then treated with 5-FU or oxaliplatin at various concentrations for 72 h. Cell viability was determined using an MTT assay. As shown in Figure 5, β-catenin shRNA treatment increased 5-FU and oxaliplatin-mediated cytotoxicity. In addition, Tcf-4 shRNA treatment resulted in a more significant increase of cytotoxicity compared to β-catenin shRNA treatment at every concentration point of 5-FU and oxaliplatin (p<0.01).

## Discussion

Colorectal cancer (CRC) is one of the most common adult malignant tumors occurring worldwide in terms of both morbidity and mortality. Despite improvements in medical therapy, the outcomes of treatment for locally advanced and metastatic disease remains disappointing, with 5-year survival rates lower than 10% in patients with metastasis. Aberrant WNT pathway signaling is an early progression event in 90% of CRC. It occurs through mutations mainly of *APC* (up to 80%) and less often of *β-catenin* (around 10%) or AXIN2 [24]. Mutations in the *APC/β-catenin* genes, which results in aberrant activation of the β-catenin/Tcf-4 pathway, are common in CRC, suggesting that targeted inhibition of this pathway could be a potential therapeutic approach to control CRC. We and others have previously reported that natural compounds and non-steroidal anti-inflammatory drugs (NSAIDs) inhibit the Wnt/β-catenin signaling pathway [22], [25], [26], [27], [28]. However, there is currently no selective inhibitor for this pathway available as a therapeutic agent. Recent studies have shown that shRNA-mediated gene silencing of β-catenin significantly inhibits cell proliferation and induces cell apoptosis in human colon cancer cells [29], [30], [31].

In the present study, we specifically focused on comparing the efficacies of β-catenin knockdown and Tcf-4 knockdown in colon cancer cells, and investigated the possible molecular mechanisms responsible for these effects. We have shown that Tcf-4 knockdown induces a significantly stronger inhibition of cell proliferation, induction of apoptosis, and enhancement of chemosensitivity in colon cancer cells compared to β-catenin knockdown.

Activated β-catenin/Tcf-4 signaling by accumulation of β-catenin in the nucleus has been implicated in human carcinogenesis, including CRC. This accumulation may result from the mutation of either the tumor suppressor gene *APC* or *β-catenin* itself. At least 60% of sporadic CRC cases contain one APC mutation, and almost half of them show abnormalities in both APC alleles [32], [33], [34]. In the present study, we conducted a detailed mechanistic study to evaluate the efficacy of inhibition of β-catenin/Tcf-4 signaling in human CRC using the human CRC SW480 cell line, which harbors mutant APC and wild-type β-catenin, and the HCT116 cell line, which harbors mutant β-catenin and wild-type APC. We first constructed adenoviral vectors carrying human β-catenin or Tcf-4 shRNA in order to knockdown the expression of β-catenin or Tcf-4. Western blot analysis revealed a dose-dependent decrease in Tcf-4 or β-catenin protein expression in SW480 and HCT116 cells at 48 h post-infection with the respective adenoviral vectors. In addition, both β-catenin shRNA and Tcf-4 shRNA markedly suppressed β-catenin/Tcf-4 transcriptional activity as well as the expression of two known target genes, Cyclin D1 and c-Myc, in SW480 and HCT116 cells. These data indicate that both β-catenin knockdown and Tcf-4 knockdown suppress the β-catenin/Tcf-4 activity *in vitro*.

Coordination between cell proliferation and apoptosis plays a very important role in the carcinogenesis and development of colon cancer. The β-catenin/Tcf-4 pathway is an important signaling pathway that tips the balance of cell proliferation and apoptosis in cancer cells. In our study, β-catenin knockdown inhibited cell proliferation and induced cell apoptosis in both SW480 and HCT116 cells. Compared to β-catenin knockdown, Tcf-4 knockdown induced a significantly greater inhibition of cell proliferation and induction of apoptosis. Furthermore, we found that Tcf-4 knockdown significantly enhanced the chemosensitivity of both SW480 and HCT116 cells to 5-FU and oxaliplatin.

We further investigated the mechanisms involved in the differential efficacies of β-catenin knockdown and Tcf-4 knockdown in SW480 and HCT116 cells. Our data showed that the protein level of FOXO4 did not change after treatment with both β-catenin and Tcf-4 shRNA. However, β-catenin shRNA was found to increase the accumulation of phosphorylated FOXO4 S193 and decrease the expression of FOXO target genes p27Kip1 and MnSOD, which is consistent with an earlier report showing that β-catenin-specific siRNA inhibited TCF reporter activity and FOXO-dependent signaling in LS174 colon cancer cells [19]. In contrast, Tcf-4 shRNA showed the opposite regulatory role to β-catenin on FOXO4. These findings suggest that β-catenin shRNA-induced down-regulation of FOXO4-dependent signaling is independent on Tcf-4.

FOXO factors play major roles in controlling cell proliferation, apoptosis, and oxidative stress [17]. [18]. Their functions are regulated by multiple signaling pathways, including AKT. In the absence of AKT activation, FOXO4 is located in the nucleus, where it functions as a transcription factor [35]. Upon AKT activation, FOXO4 becomes phosphorylated on three highly conserved serine and threonine residues (Ser-193, Thr-28, and Ser-258) followed by inactivation and nuclear exclusion [36]. Essers et al. [19] showed that there was functional interaction between β-catenin and FOXO in oxidative stress signaling, and β-catenin-specific shRNA reduced TCF and FOXO transcriptional activity in LS174T colon cancer cells, which express a mutant form of β-catenin. Accumulating evidence suggests that FOXO factors function as tumor suppressors in a variety of cancers. Tang et al. [37] also found that FOXO4 inhibited the expression of HIF-1α and VEGF in cancer cells. Moreover, a recent study showed that overexpression of wild-type FOXO4 led to an increase in doxorubicin-mediated cytotoxicity in HCT116 colon cancer cells, which was further exacerbated by overexpression of a FOXO4 mutant localized exclusively in the nucleus [38].

Therefore, we hypothesize that the upregulation of FOXO4 activity is responsible for the greater efficacy of Tcf-4 shRNA effects in colon cancer cells compared to β-catenin shRNA. On the one hand, Tcf-4 and FOXO4 may be competing for β-catenin [19]. When Tcf signaling is inhibited, β-catenin binds directly to FOXO4 and enhances FOXO4 transcriptional activity. On the other hand, Tcf4 knockdown may downregulate certain signaling pathways, such as Akt. A recent report shows that a significant reduction of AKT2 levels and phosphorylation of Akt was detected after knockdown of Tcf-4 using Tcf-4 siRNA in glioma cells [39]. Therefore, inactivation of Akt by Tcf-4 shRNA results in dephosphorylation of FOXO4, which consequently activates FOXO-dependent signaling.

In conclusion, we have shown that targeted inhibition of β-catenin/Tcf-4 signaling inhibits cell proliferation, induces apoptosis, and enhances chemosensitivity of colon cancer cells. In addition, compared to β-catenin knockdown, Tcf-4 knockdown showed greater efficacy for inhibiting CRC cell growth and inducing apoptosis, which may be related to an increase in FOXO4 transcriptional activity. These results suggest that Tcf-4 may be an attractive therapeutic target for CRC therapy. Future studies are needed to confirm these findings and ascertain the utility of targeting Tcf-4.
